# Identification of Gene Clusters Associated with Host Adaptation and Antibiotic Resistance in Chinese *Staphylococcus aureus* Isolates by Microarray-Based Comparative Genomics

**DOI:** 10.1371/journal.pone.0053341

**Published:** 2013-01-07

**Authors:** Henan Li, Chunjiang Zhao, Hongbin Chen, Feifei Zhang, Wenqiang He, Xiaojuan Wang, Qi Wang, Ruifu Yang, Dongsheng Zhou, Hui Wang

**Affiliations:** 1 Department of Clinical Laboratory, Peking University People’s Hospital, Beijing, People’s Republic of China; 2 State Key Laboratory of Pathogen and Biosecurity, Beijing Institute of Microbiology and Epidemiology, Beijing, People’s Republic of China; Ghent University, Belgium

## Abstract

A comparative genomic microarray comprising 2,457 genes from two whole genomes of *S. aureus* was employed for the comparative genome hybridization analysis of 50 strains of divergent clonal lineages, including methicillin-resistant *S. aureus* (MRSA), methicillin-susceptible *S. aureus* (MSSA), and swine strains in China. Large-scale validation was confirmed via polymerase chain reaction in 160 representative clinical strains. All of the 50 strains were clustered into seven different complexes by phylogenetic tree analysis. Thirteen gene clusters were specific to different *S. aureus* clones. Ten gene clusters, including seven known (vSa3, vSa4, vSaα, vSaβ, Tn5801, and phage ϕSa3) and three novel (C8, C9, and C10) gene clusters, were specific to human MRSA. Notably, two global regulators, *sarH2* and *sarH3*, at cluster C9 were specific to human MRSA, and plasmid pUB110 at cluster C10 was specific to swine MRSA. Three clusters known to be part of SCCmec, vSa4 or Tn5801, and vSaα as well as one novel gene cluster C12 with homology with Tn554 of *S. epidermidis* were identified as MRSA-specific gene clusters. The replacement of ST239-*spa* t037 with ST239-*spa* t030 in Beijing may be a result of its acquisition of vSa4, phage ϕSa1, and ϕSa3. In summary, thirteen critical gene clusters were identified to be contributors to the evolution of host specificity and antibiotic resistance in Chinese *S. aureus*.

## Introduction

*Staphylococcus aureus* is an opportunistic pathogen and the major causative agent of numerous hospital- and community-acquired infections in humans. It is also a common causative agent of animal infections. The major MRSA clones that cause infectious diseases worldwide are reported to belong to only a few pandemic lineages. In China, the most common human MRSA lineages belong to ST239 and ST5 [1]. Meanwhile, ST9 was identified as the dominant swine MRSA lineage in China [2]. *S. aureus* contains many types of genomic islands including plasmids, transposons (Tn), insertion sequences (IS), bacteriophages, pathogenic islands, and staphylococcal cassette chromosomes. These elements play a central role in the pathogen’s adaptation process to different stresses, and are means to transfer genetic information among and within bacterial species [3]. Each *S. aureus* lineage carries a unique combination of genomic islands. In the genome of *S. aureus* Mu50, nine genomic islands have been identified, including vSa3, vSa4, vSaα, vSaβ, vSaγ, SCC*mec*, phage ϕSa1, phage ϕSa3, and Tn5801 [4]. The carriage of genomic islands in *S. aureus* can alter the pathogenic and resistance potential of the strains. The dissemination of particular clones in a specific environment or host in favor of other strains, or the replacement of clones in a single environment suggests a genetic basis for epidemics related to genomic islands. This has fuelled efforts to identify novel genomic islands associated with the evolution of antibiotic resistance and host adaptation in Chinese *S. aureus.*

Comparative genome hybridization (CGH) is an efficient method to identify critical gene clusters. When applied to pathogenic *S. aureus*, CGH unveils the variability in terms of gene content in regions related to pathogenicity and gives new insights into the evolutionary aspects of *S. aureus*. The high discriminatory power of this technique has been used to distinguish major MRSA lineages, community-associated MRSA strains, and predominant *S. aureus* lineages [5], [6], [7], [8].

This study aimed to compare the genetic repertoire of different *S. aureus* clones through microarray-based comparative genomics to identify the gene clusters that may explain the evolutionary mystery of *S. aureus*: (i) Many articles reported that human MRSA may originate in animals [9], but host-specific genes or gene clusters were rarely found. (ii) MSSA showed more diverse patterns compared with the relative preponderance of a few MRSA clones. (iii) ST239 and ST5 were the most predominant MRSA clones in China [1]. From 1994 to 2000 in Beijing, ST239-*spa* t030 rapidly replaced t037 and became the major MRSA clone [10]. In this study, we identified 13 gene clusters in the *S. aureus* genome associated with the evolution of antibiotic resistance and host specificity by using CGH microarray. The gene clusters were confirmed by large-scale validation via polymerase chain reaction (PCR) in 160 clinical strains. Among these clusters, several critical genes and four novel gene clusters related to the evolution of resistance and host specificity in Chinese *S. aureus* have not yet been reported.

## Results

### Overall Genome Diversity in *S. aureus*

The microarray comprised all the genetic information found in only two *S. aureus* genomes, Mu50 and CN79. CGH microarray analysis revealed extensive genome diversity within the *S. aureus* species. Within the 2,457 genes present on the *S. aureus* microarray, all of the 50 strains shared 1,738 genes (70.7%) and 719 (29.3%) genes were absent in at least one strain. An average of 260 (10.6%) genes were absent per strain compared to the genes present on the microarray.

**Figure 1:**
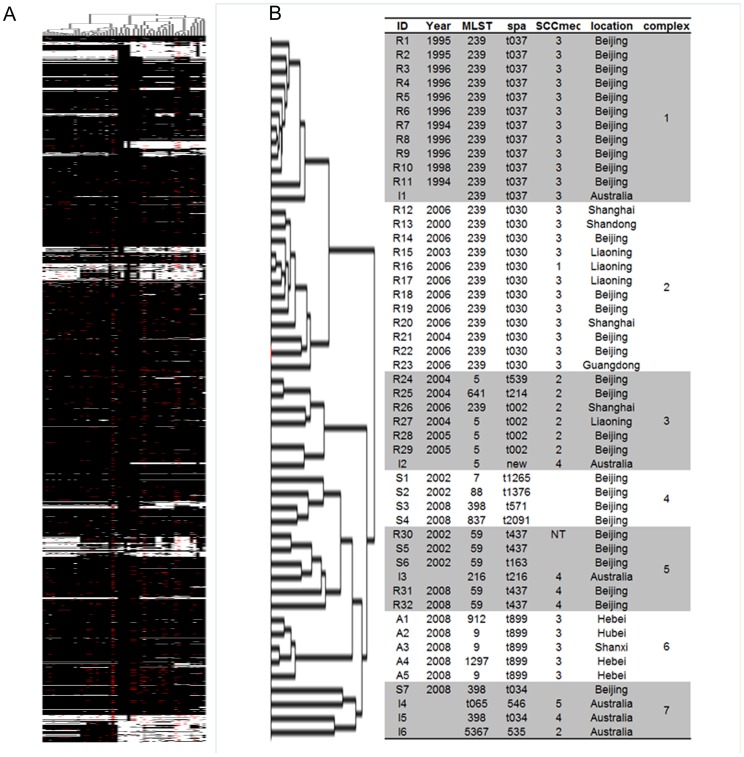
Cluster analysis of 50 strains via microarray. (A) Phylogenetic tree analysis of 50 strains. White bars: gene absence, black bars: gene presence, red bars: no information. (B) Characteristics of 50 strains. Cluster analysis indicated that all of the 50 strains were clustered into seven different complexes.

Cluster analysis indicated that all of the 50 strains were clustered into seven different complexes (Fig. 1). Strains in the same complex showed similar backgrounds such as isolation time, location, species, and lineage. Different complexes represented different backgrounds. Complex 1 included 11 MRSA (ST239-*spa* t037) isolated in Beijing before 2000. Complex 2 included 12 MRSA (ST239-*spa* t030) isolated from 2000 to 2006. Complex 3 included 7 MRSA and mostly ST5. All of the strains in Complex 4 were MSSA. Complex 5 included 3 ST59-MRSA and 2 ST59-MSSA. Swine MRSA were clustered in complex 6. The Australian strains were scattered, and 3 out of 6 were in complex 7.

### Comparative Genomics of Human and Swine *S. aureus* Strains

**Figure 2:**
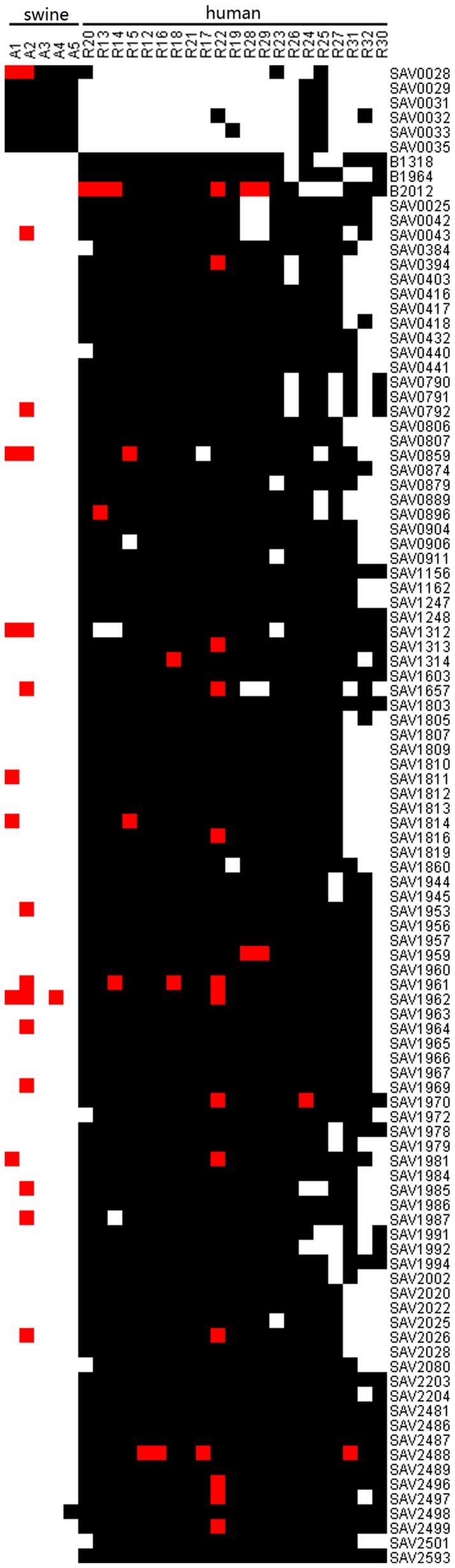
Genes associated with host specific. A total of 96 genes were present in greater than 80% human MRSA while 6 genes were present in all swine MRSA. White squares: gene absence, black squares: gene presence, red squares: no information.

CGH microarray was used to study human- and swine-derived MRSA at the genomic level. A total of 1,851 genes were present in both human and swine strains. A total of 102 genes were associated with host specificity, specifically in human or swine MRSA (Fig. 2). Among these genes, 96 genes were present in greater than 80% human MRSA while 6 genes were present in all swine MRSA. Host-specific genes contained 56 pathogenicity island genes (3 in vSa3, 5 in vSa4, 2 in Tn5801, 6 in vSaα, 10 in vSaβ, 2 in vSaγ, and 28 in phage ϕSa3), 10 phage-related genes, 4 resistant-related genes (fmhC, mecR1, mecI, and lytN) [11], 2 global regulators (sarH2 and sarH3), 5 transposes, 2 helicases, and 24 hypothetical proteins.

**Figure 3:**
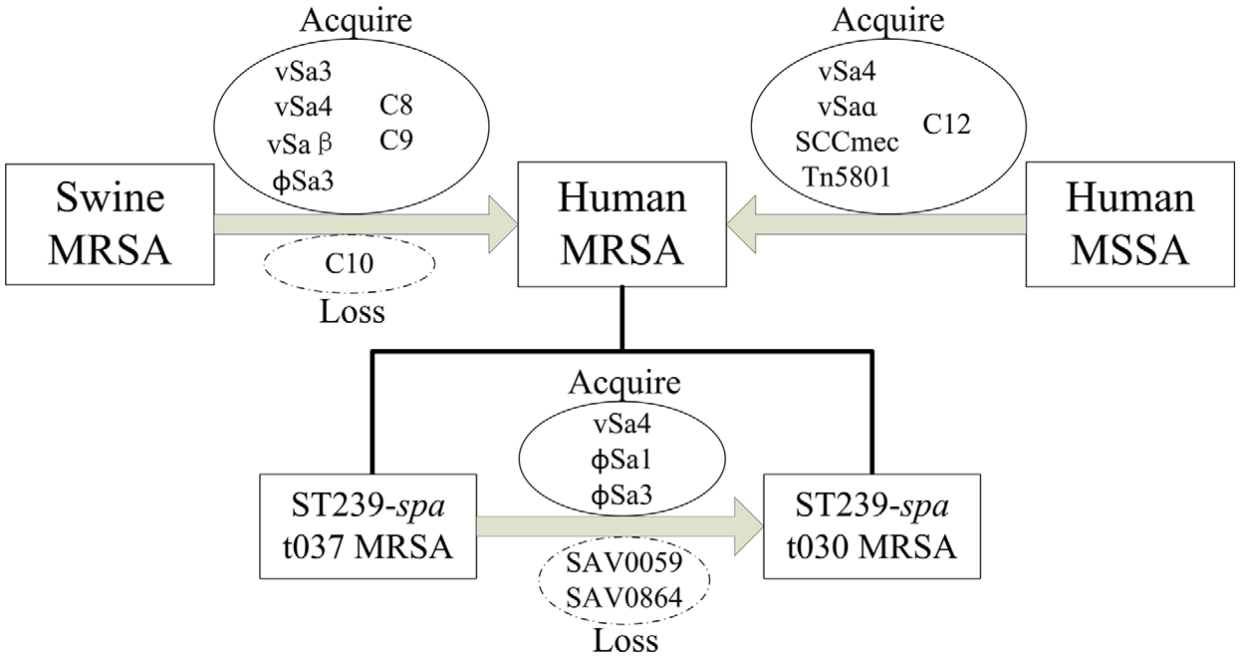
Evolutionary ideograph of genomic islands in the evolution of *S. aureus.*

Among these genes, several continuous genes formed 10 clusters (i.e. more than three contiguous genes) (Table 1 and Fig. 3). Seven clusters belonged to known genomic islands vSa3, vSa4, vSaα, vSaβ, Tn5801, and phage ϕSa3. Human-specific genomic island phage ϕSa3 contained immune evasion complex genes that encode the staphylokinase (*sak*). This prophage, integrated into β-hemolysin locus, has been found in most isolates infecting humans but not animals [12]. Human-specific genomic island vSaβ included six virulence genes, namely, *splA, splB, splC, splD*, *splF*, and *lukD*, which enhanced the virulence of MRSA and facilitated human infection. In addition, type I restriction modification (R-M) system gene *hsdS* (SAV0432 and SAV1807) were identified in human-specific genes, which confirmed their function in regulating gene horizontal transfer. Three gene clusters (C8, C9 and C10) were distinct from any known genomic islands. Cluster C8 (SAV1312–SAV1314) contained three function-unknown genes. Cluster C9 (SAV2481–SAV2499) carried two global regulators, *sarH2* and *sarH3*, indicating its potential regulatory function in host specificity. Swine-specific cluster C10 (SAV0028-SAV0035) belonging to plasmid pUB110 contained the resistance gene *aadD*.

**Table 1:** Identification of gene clusters by microarray-based comparative genomics.

Cluster No.	Cluster range	Size	Location	ST239-*spa* t030 MRSA specific cluster^a^	Antibiotic resistancecluster^b^	Host specificity cluster^c^
C1	SAV1944–SAV1969	∼23 kb	ϕSa3	+		+
C2	SAV1979–SAV1987	∼6 kb	ϕSa3	+		+
C3	SAV2018–SAV2028	∼8 kb	vSa4	+	+	+
C4	SAV0392–SAV0418	∼28 kb	Tn5801 and vSaα		+	+
C5	SAV0790–SAV0792	∼3 kb	vSa3			+
C6	SAV1809–SAV1814	∼5 kb	vSaβ			+
C7	SAV1991–SAV1994	∼2 kb	ϕSa3			+
C8	SAV1312–SAV1314	∼2 kb	Novel gene cluster			+
C9	SAV2481–SAV2499	∼19 kb	Novel gene cluster			+
C10	SAV0028–SAV0035	∼4 kb	Novel gene cluster			+
C11	SAV0038–SAV0057	∼19 kb	SCCmec		+	
C12	SAV1653–SAV1659	∼6 kb	Novel gene cluster		+	
C13	SAV0885–SAV0904	∼18 kb	ϕSa1	+		

a Comparison between ST239-spa t037 before 2000 and ST239-spa t030 after 2000 in Beijing.

b Comparison between human MRSA and MSSA.

c Comparison between human MRSA and swine MRSA.

The representative genes of 10 clusters associated with host specificity were further analyzed in 76 human MRSA and 20 swine MRSA strains. The presense of representative genes showed significant differences between human and swine MRSA strains (Table 2, *P*<0.005). Interestingly, not all human MRSA carried human-specific gene clusters identified by our CGH microarray. ST59 MRSA did not carry human-specific clusters C3, C4, and C6, and showed a similar pattern as swine MRSA.

**Table 2:** PCR validation results of gene cluster associated with host specificity.

Cluster No.^a^	Representative gene		Number of positive isolates (%)		*P* value
			Human MRSA	Swine MRSA	
	Gene symbol	Gene description	N = 76	N = 20	
C1	SAV1948	*enterotoxin P*	67 (88.2)	1 (5)	<0.0001
C2	SAV1979	*phi PVL ORF 50-like protein*	49 (64.5)	1 (5)	<0.0001
C3	SAV2022	*hypothetical protein*	50 (65.8)	0	<0.0001
C4	SAV0398	*tetM (Tn5801)*	64 (84.2)	0	<0.0001
C5	SAV0791	*hypothetical protein*	58 (76.3)	0	<0.0001
C6	SAV1813	*splA*	35 (46.1)	2 (10)	0.003
C7	SAV1994	*anti repressor*	71 (93.4)	2 (10)	<0.0001
C8	SAV1313	*hypothetical protein*	76 (100)	1 (5)	<0.0001
C9	SAV2498	*sarH3*	73 (96.1)	1 (5)	<0.0001
C10	SAV0031	*pre*	3 (21.4)	18 (90)	<0.0001

a Clusters that are not listed were not associated with host specificity.

### Comparative Genomics of MRSA and MSSA Strains

MSSA showed more diverse patterns compared with the relative preponderance of a few MRSA clones. The differences between 6 MSSA and 18 MRSA isolated in China were analyzed, and 75 genes were found to be more frequently present in MRSA. Among these genes, 38 are located at known genomic islands, including SCCmec, vSa4, vSaα and Tn5801 (Table 1 and Fig. 3), which contained several resistance genes such as *mecA*, *ermA*, *tetM*, and *ant(9)*. We also identified a novel MRSA-specific gene cluster C12, which contained 2 resistance genes [*ermA* and *ant(9)*], 3 transposase genes, and 1 function-unknown genes. Sequence alignment indicated that C12 had homology with Tn554 of *S. epidermidis* and may represent a novel resistant island.

To determine whether the resistance genes in MRSA isolates differed between lineages, 10 resistance genes in all of the MRSA strains analyzed by microarray were compared (Table 3). The *mecA*, *aphA*, *dfrA*, *isaA*, *msrA*, and *msrB* were detected in all of the analyzed isolates. The *ermA* and *tetM* were absent in human ST59, swine ST9, and several ST5 strains. The *ant (9)* gene was detected in various lineages: ST239 (n = 20), ST59 (n = 2), and swine ST9 (n = 1), was but absent in all of the ST5 strains. The *aadD* gene was specific to all of the swine ST9 strains and single ST5 isolate, but absent in the ST239 and ST59 strains. In summary, the ST239 and ST5 MRSA isolates display considerable antimicrobial resistance genotype diversity, with ST239 and ST5 being the most predominant clones in China [1].

**Table 3:** Comparison of the distribution of selected resistance genes among different lineages of MRSA strains analyzed in CGH microarray.

Resistance gene	Gene description	Cluster No.	Number of positive isolates (%)			
			ST239	ST5	ST59	ST9
			N = 24	N = 5	N = 3	N = 5
*mecA*	*penicillin binding protein 2 prime*	*C11*	24 (100)	3 (60)	2 (66.7)	5 (100)
*aadD*	*kanamycin nucleotidyltransferase*	*C10*	0	1 (20)	0	5 (100)
*aphA*	*phosphotransferase A*	*–* a	24 (100)	5 (100)	3 (100)	5 (100)
*ermA*	*rRNA methylase*	*C11, C12*	24 (100)	2 (40)	0	0
*tetM*	*tetracycline resistance protein*	*C4*	24 (100)	2 (40)	0	0
*dfrA*	*dihydrofolate reductase*	*–*	24 (100)	5 (100)	3 (100)	5 (100)
*ant(9)*	*O-nucleotidylltransferase*	*C11, C12*	20 (83.3)	0	2 (66.7)	1 (20)
*isaA*	*immunodominant antigen A*	*–*	24 (100)	5 (100)	3 (100)	5 (100)
*msrA*	*methionine sulfoxide reductase A*	*–*	24 (100)	5 (100)	3 (100)	5 (100)
*msrB*	*methionine sulfoxide reductase B*	*–*	24 (100)	5 (100)	3 (100)	5 (100)

a Resistance genes were not located at the reported gene clusters.

To further validate these gene clusters, 48 clinical MRSA and 48 MSSA strains were analyzed by detecting representative genes in these clusters. PCR validation results showed that four representative genes of clusters were detected in most MRSA, but were absent in most MSSA strains (Table 4, *P*<0.0001).

**Table 4:** PCR validation results of gene clusters associated with antibiotic resistance.

Cluster No.^a^	Representative gene		Number of positive isolates (%)		*P value*
			MRSA	MSSA	
	Gene symbol	Gene description	N = 48	N = 48	
C3	SAV2022	hypothetical protein	25 (52.1)	6 (12.5)	<0.0001
C4	SAV0398	*tetM*	41 (85.4)	0	<0.0001
C11	SAV0052	*ermA*	42 (87.5)	4 (8.3)	<0.0001
C12	SAV1656	*ant (9)*	35 (72.9)	6 (12.5)	<0.0001

a Clusters that are not listed were not associated with antibiotic resistance.

### Comparative Genomics of Predominant MRSA Clones in China

From 1994 to 2000 in Beijing, the most predominant MRSA clone was ST239-*spa* t037. Since 2000, ST239-*spa* t030 has rapidly replaced t037 and has become the major clone [10]. A comparison of 11 *spa* t037 and 5 *spa* t030 genome information showed that 309 variable genes were variable in 16 strains. Ninety-eight genes are more frequent in *spa* t030, and 2 genes (SAV0059 and SAV0864) are more frequent in *spa* t037. Fifty-four pathogenic island genes (10 in vSa4, 8 in vSa3, 4 in vSaα, and 30 in phage ϕSa3), 12 phage-related genes, and 1 transcription-related gene were included in these genes. For ST239-*spa* t030 specific genes, four gene clusters (Table 1) may contribute to the MRSA evolution from ST239-*spa* t037 to ST239-*spa* t030 (Fig. 3). These gene clusters belonged to previously characterized genomic islands vSa4, phage ϕSa1, and ϕSa3. Notably, phage ϕSa3 was unique to ST239- *spa* t030 MRSA and carried two toxin genes, *sak (staphylokinase)* and *sep (enterotoxin P),* which may contribute to its increased virulence and epidemiology [13]. Besides, most variable genes found in these islands have unknown functions.

Except for ST239, ST5 was the second predominant clone in China. We analyzed the presence of antibiotic-resistant clusters via large-scale PCR validation in 43 ST239 or ST5 MRSA strains and 5 ST59 MRSA strains. Greater than 60% of the predominant clones ST239 and ST5 existed these antibiotic-resistant clusters, but none of ST59 strains existed these clusters. The carriage of multiple antibiotic resistance gene clusters probably enhanced the adaptability and competitiveness of ST239 and ST5, as well as contributed to the prevalence in China.

## Discussion

Extensive genetic variations were identified among 50 strains representing the major dominant lineages of *S. aureus* from human or swine in China by microarray-based comparative genomic. Within the 2,457 genes present on the *S. aureus* microarray, 1,738 genes (70.7%) were present in all of the *S. aureus* strains studied, suggesting that these genes were essential for *S. aureus* maintenance. Conversely, 29.3% of *S. aureus* genes were strain-specific. Some of these genes encoded genomic islands that facilitate the colonization of specialized host or antibiotic resistance.

The carriage of genomic islands in *S. aureus* has the ability to alter the pathogenic- and resistance-potential of strains [3]. Overall, each *S. aureus* lineage carried a unique combination of genomic islands. Genomic comparison of the different complexes revealed 13 gene clusters (Table 1). Among these clusters, vSa3, vSa4, vSaα, vSaβ, phage ϕSa1, phage ϕSa3, SCCmec, and Tn5801 have been identified [4]. These genomic islands carried approximately one-half of the *S. aureus* toxins or virulence factors, and the variation of these genes contributed to the pathogenic potential of this species [14]. Meanwhile, four novel gene clusters that have not been reported before were notably revealed.

Previous studies identified that phage ϕSa3 was more common in human isolates than in animal isolates [6]. The phage ϕSa3 encoded *scin*, *chip,* and/or *sak* was involved in the host immune evasion and was proven to interact specifically with the human immune system [15]. In our research, genomic islands vSa3, vSa4, vSaα, and vSaβ, as well as two novel gene clusters (C8 and C9) were also associated with human specificity [16]. In particular, type I R-M system gene *hsdS* was located at vSaα, vSaβ, and global regulators, *sarH2* and *sarH3* at C9. *SarH2*, also known as *sarU*, is *sarA* homolog, which is repressed by *sarH3* (also known as *sarT*) and regulates virulence genes in *S. aureus* [17]. The two global regulators possibly enhance the regulatory efficiency of MRSA in human infection. Further investigation of these regulators is necessary.

SCCmec, Tn5801, vSaα, vSa4, and a novel gene cluster were more frequently present in MRSA than in MSSA. These gene clusters contained abundant resistance genes [*mecA*, *tetM*, *ermA*, and *ant(9)*] that increased the virulence and resistance of MRSA [18]. Novel gene cluster C12 associated with resistance was similar to Tn554 of *S. epidermidis* by sequence alignment, which may transfer from *S. epidermidis*. Tn554 containing *ermA* gene was related to macrolides-lincosamides-streptogramin B resistance [19].

ST239 and ST5 were the most predominant MRSA clones in China. From 1994 to 2008 in Beijing, ST239-*spa* t030 rapidly replaced t037 and became the major MRSA clone [10]. In this study, vSa4, phage ϕSa1, and phage ϕSa3 were found to be unique to ST239-*spa* t030 and carried two toxin genes, *sak* and *sep*, that may contribute to its increased virulence and rapid replacement of ST239-*spa* t037 [13]. Meanwhile, large-scale validation indicated that the two major epidemic clones, ST239 and ST5 MRSA, display considerable antimicrobial resistance genotype diversity that contributes to the prevalence in China.

Comparative analysis of *S. aureus* suggested variations in the evolutionary history of genomic islands [20]. The movement of these genomic islands may enable *S. aureus* to evolve and grow through the acquisition of virulence and resistance genes. Clearly, horizontal gene transfer has played a fundamental role in the evolution of pathogenic *S. aureus*, particularly by the assortive recombination of genomic islands containing virulence and antibiotic resistance genes. Several genomic islands distributed within certain lineages at a higher frequency than others, suggesting some barriers to the successful horizontal transfer of genomic islands. A general barrier to horizontal gene transfer in *S. aureus* is the R-M system [21]. The role of the R-M system is to prevent the uptake of potentially harmful or lethal DNA such as bacteriophage that lyses and kills bacteria or to prevent the acquisition of superfluous genes that may compromise fitness due to the metabolic demand associated with their expression [22]. In our studies, the type I R-M system gene *hsdS* varied significantly between the strains of different complexes. Therefore, different strains with different horizontal gene transfer abilities resulted in the epidemiology of specific clones.

CGH is an efficient method to identify novel genomic islands. The microarray comprised all the genetic information found in only two *S. aureus* genomes, Mu50 and CN79. However, if isolates with specific biological characteristics were analyzed with these microarrays, this specific genetic information will most likely not be detected. The way to entirely exclude this problem would be to sequence all of the strains, which will help us to understand the detailed information of genomic islands identified in this study.

In brief, our study provided an overview of the genome diversity present in *S. aureus* in China, an important human and animal pathogen worldwide. The microarray-based comparative genomic analysis clarified the functions of known genomic islands and four novel gene clusters in the evolution of antibiotic resistance and host adaptation in Chinese *S. aureus*. Further investigation on the gene cluster functions is necessary.

## Materials and Methods

### Ethics Statement

All human *S. aureus* strains were collected from Gram-Positive Cocci Resistance Surveillance and Chinese Antimicrobial Resistance Surveillance of Nosocomial infections program. Both of the Surveillance programs were approved by the Medical Ethical Committee of Peking University People’s hospital. Swine strains and Australian MRSA strains were kindly provided by Dr. Yue Ma and Dr. Fanrong Kong. Collecting animal samples was in accordance with the Chinese Law on Animal Health and Welfare.

### Bacterial Strains

A total of 50 *S. aureus* strains were used, including 45 human and 5 swine strains. From 1994 to 2008, 39 human *S. aureus* strains were collected from 5 provinces (Beijing, Guangdong, Liaoning, Shandong, and Shanghai) in China, including 11 ST239-*spa* t037 MRSA, 12 ST239-*spa* t030 MRSA, 4 ST5 MRSA, 3 ST59 MRSA, and 7 MSSA. Five swine strains collected from swine in ShanXi, HeBei, and HuBei provinces in 2008 [2] were kindly given from Dr. Yue Ma. The strains were selected to cover the major dominant lineages of *S. aureus* from humans and swines in China, and to represent the sufficient abundance of *S. aureus* populations in China. Meanwhile, six Australian MRSA strains kindly provided by Dr. Fanrong Kong were selected for comparisons.

### Microarray Design

A total of 2,457 open reading frames (genes) were amplified from the whole-genome *S. aureus* sequenced strains Mu50 and CN79 by PCR using gene-specific primers [23]. *S. aureus* CN79 isolated from blood was determined as heterogeneous vancomycin-intermediate *S. aureus* by a population analysis profile (PAP)-area under the curve (AUC) method; it belonged to the Chinese predominant clone ST239-*spa* t030 MRSA [24]. The purified PCR products were spotted in duplicate on CSS-1000 silylated glass slides (CEL) using a SpotArray72 microarray printing system (Perkin-Elmer Life Sciences, Massachusetts, USA) to construct the DNA microarrays.

### Microarray Labeling, Hybridizations, and Scanning

Genomic DNAs were extracted using conventional sodium dodecyl sulfate lysis and phenol-chloroform extraction method. A mixture of equal quantities of Mu50 and CN79 genomic DNAs was used as reference DNA. Purified PCR products were referred to as the tested DNA. Cy3- or Cy5-labeled probes were generated by priming the reference or test DNA with random hexamers and extension with Klenow polymerase. The labeled reference and test DNAs were combined to hybridize with the microarrays by dual-fluorescence hybridization. The hybridized slides were scanned using a GenePix 4100A personal microarray scanner (Axon Instruments, Foster City, California, USA.). The scanning images were processed, and the data were further analyzed using GenePix Pro 5.0 software (Axon Instruments, Foster City, California, USA) combined with Microsoft Excel software.

### Microarray Data Analysis

Spots with signal intensity (median) in the channel of the reference DNA less than two folds of the local background intensity (median) were rejected from further analysis. Spots with bad data because of slide abnormalities were discarded as well. Data normalization was performed on the remaining spots using total intensity normalization methods. A ratio of intensity (Test DNA normalized intensity/Reference DNA normalized intensity) was recorded for each spot and then converted to log2. Genes with fewer than three data points were considered unreliable and were accordingly removed. The averaged log2 ratio for each remaining gene on the two replicate slides was ultimately calculated. If 20% of the strains had a gene with missing data, the gene was removed. A total of 2,457 genes were included in the final dataset. A log2 value equal to or lower than −1 was used to define the absence of a gene in a given strain. The microarray data had been deposited in public database ArrayExpress (Accession NO.: A-MEXP-2250).

### Clustering and Phylogenetic Analysis

The final absent (0) or present (1) was assigned to each gene for each strain in the CGH data,. Hierarchical clustering of gene expression across species was performed with Cluster 3.0 using the uncentered Pearson correlation as the distance metric [25]. The clustered microarray data were displayed by the TreeView tool.

### PCR Validation

The selected representative genes by CGH analysis were confirmed via gene-specific PCR. The primers used, listed in Table S1, were the same ones used to generate PCR products spotted on our microarray. PCR products were amplified with the following conditions: 94°C for 5 min, followed by 30 cycles of 94°C for 30 s, 60°C for 30 s, 72°C for 60 s, and a final elongation step of 72°C for 5 min. 4 µL of each reaction was run on a 1% agarose gel. A positive reaction was recorded if a single clear band with the correct size was present.

### Statistical Analysis

Statistical analysis was carried out using Statistical Package for Social Sciences 14.0 for Windows (SPSS). For statistical analysis, χ^2^ test or Fisher’s exact test was used to analyze the results. A *P* value of <0.05 was considered statistically significant.

## Supporting Information

Table S1 **Primers of representative genes used for PCR validation.**
(DOC)

## References

[1] Liu Y, Wang H, Du N, Shen E, Chen H, et al (2009) Molecular evidence for spread of two major methicillin-resistant *Staphylococcus aureus* clones with a unique geographic distribution in Chinese hospitals. Antimicrob Agents Chemother 53: 512–518. 19029328 10.1128/AAC.00804-08 PMC2630620

[2] Cui S, Li J, Hu C, Jin S, Li F, et al (2009) Isolation and characterization of methicillin-resistant *Staphylococcus aureus* from swine and workers in China. J Antimicrob Chemother 64: 680–683. 19684078 10.1093/jac/dkp275

[3] Lindsay JA (2010) Genomic variation and evolution of *Staphylococcus aureus* . Int J Med Microbiol 300: 98–103. 19811948 10.1016/j.ijmm.2009.08.013

[4] Gill SR, Fouts DE, Archer GL, Mongodin EF, Deboy RT, et al (2005) Insights on evolution of virulence and resistance from the complete genome analysis of an early methicillin-resistant *Staphylococcus aureus* strain and a biofilm-producing methicillin-resistant *Staphylococcus epidermidis* strain. J Bacteriol 187: 2426–2438. 15774886 10.1128/JB.187.7.2426-2438.2005 PMC1065214

[5] Jamrozy DM, Fielder MD, Butaye P, Coldham NG (2012) Comparative Genotypic and Phenotypic Characterisation of Methicillin-Resistant *Staphylococcus aureus* ST398 Isolated from Animals and Humans. PLoS One 7: e40458. 22792335 10.1371/journal.pone.0040458 PMC3394705

[6] Sung JM, Lloyd DH, Lindsay JA (2008) *Staphylococcus aureus* host specificity: comparative genomics of human versus animal isolates by multi-strain microarray. Microbiology 154: 1949–1959. 18599823 10.1099/mic.0.2007/015289-0

[7] Yamamoto T, Takano T, Higuchi W, Iwao Y, Singur O, et al (2012) Comparative genomics and drug resistance of a geographic variant of ST239 methicillin-resistant *Staphylococcus aureus* emerged in Russia. PLoS One 7: e29187. 22276107 10.1371/journal.pone.0029187 PMC3261861

[8] Christianson S, Golding GR, Campbell J, Mulvey MR (2007) Comparative genomics of Canadian epidemic lineages of methicillin-resistant *Staphylococcus aureus* . J Clin Microbiol 45: 1904–1911. 17428941 10.1128/JCM.02500-06 PMC1933033

[9] Guinane CM, Ben ZN, Tormo-Mas MA, Weinert LA, Lowder BV, et al (2010) Evolutionary genomics of *Staphylococcus aureus* reveals insights into the origin and molecular basis of ruminant host adaptation. Genome Biol Evol 2: 454–466. 20624747 10.1093/gbe/evq031 PMC2997551

[10] Chen H, Liu Y, Jiang X, Chen M, Wang H (2010) Rapid change of methicillin-resistant *Staphylococcus aureus* clones in a Chinese tertiary care hospital over a 15-year period. Antimicrob Agents Chemother 54: 1842–1847. 20176895 10.1128/AAC.01563-09 PMC2863666

[11] van Hoek AH, Mevius D, Guerra B, Mullany P, Roberts AP, et al (2011) Acquired antibiotic resistance genes: an overview. Front Microbiol 2: 203. 22046172 10.3389/fmicb.2011.00203 PMC3202223

[12] van Wamel WJ, Rooijakkers SH, Ruyken M, van Kessel KP, van Strijp JA (2006) The innate immune modulators staphylococcal complement inhibitor and chemotaxis inhibitory protein of *Staphylococcus aureus* are located on beta-hemolysin-converting bacteriophages. J Bacteriol 188: 1310–1315. 16452413 10.1128/JB.188.4.1310-1315.2006 PMC1367213

[13] Chambers HF, Deleo FR (2009) Waves of resistance: *Staphylococcus aureus* in the antibiotic era. Nat Rev Microbiol 7: 629–641. 19680247 10.1038/nrmicro2200 PMC2871281

[14] Malachowa N, DeLeo FR (2010) Mobile genetic elements of *Staphylococcus aureus* . Cell Mol Life Sci 67: 3057–3071. 20668911 10.1007/s00018-010-0389-4 PMC2929429

[15] de Haas CJ, Veldkamp KE, Peschel A, Weerkamp F, Van Wamel WJ, et al (2004) Chemotaxis inhibitory protein of *Staphylococcus aureus*, a bacterial antiinflammatory agent. J Exp Med 199: 687–695. 14993252 10.1084/jem.20031636 PMC2213298

[16] Baba T, Bae T, Schneewind O, Takeuchi F, Hiramatsu K (2008) Genome sequence of *Staphylococcus aureus* strain Newman and comparative analysis of staphylococcal genomes: polymorphism and evolution of two major pathogenicity islands. J Bacteriol 190: 300–310. 17951380 10.1128/JB.01000-07 PMC2223734

[17] Manna AC, Cheung AL (2003) sarU, a sarA homolog, is repressed by SarT and regulates virulence genes in *Staphylococcus aureus* . Infect Immun 71: 343–353. 12496184 10.1128/IAI.71.1.343-353.2003 PMC143423

[18] Deurenberg RH, Stobberingh EE (2009) The molecular evolution of hospital- and community-associated methicillin-resistant *Staphylococcus aureus* . Curr Mol Med 9: 100–115. 19275621 10.2174/156652409787581637

[19] Tillotson LE, Jenssen WD, Moon-McDermott L, Dubin DT (1989) Characterization of a novel insertion of the macrolides-lincosamides-streptogramin B resistance transposon Tn554 in methicillin-resistant *Staphylococcus aureus* and *Staphylococcus epidermidis* . Antimicrob Agents Chemother 33: 541–550. 2543284 10.1128/aac.33.4.541 PMC172476

[20] Fitzgerald JR, Sturdevant DE, Mackie SM, Gill SR, Musser JM (2001) Evolutionary genomics of *Staphylococcus aureus*: insights into the origin of methicillin-resistant strains and the toxic shock syndrome epidemic. Proc Natl Acad Sci U S A 98: 8821–8826. 11447287 10.1073/pnas.161098098 PMC37519

[21] Murray NE (2000) Type I restriction systems: sophisticated molecular machines (a legacy of Bertani and Weigle). Microbiol Mol Biol Rev 64: 412–434. 10839821 10.1128/mmbr.64.2.412-434.2000 PMC98998

[22] Ender M, McCallum N, Adhikari R, Berger-Bachi B (2004) Fitness cost of SCCmec and methicillin resistance levels in *Staphylococcus aureus* . Antimicrob Agents Chemother 48: 2295–2297. 15155238 10.1128/AAC.48.6.2295-2297.2004 PMC415608

[23] Kuroda M, Ohta T, Uchiyama I, Baba T, Yuzawa H, et al (2001) Whole genome sequencing of meticillin-resistant *Staphylococcus aureus* . Lancet 357: 1225–1240. 11418146 10.1016/s0140-6736(00)04403-2

[24] Sun W, Chen H, Liu Y, Zhao C, Nichols WW, et al (2009) Prevalence and characterization of heterogeneous vancomycin-intermediate *Staphylococcus aureus* isolates from 14 cities in China. Antimicrob Agents Chemother 53: 3642–3649. 19546358 10.1128/AAC.00206-09 PMC2737858

[25] Wohlbach DJ, Kuo A, Sato TK, Potts KM, Salamov AA, et al (2011) Comparative genomics of xylose-fermenting fungi for enhanced biofuel production. Proc Natl Acad Sci U S A 108: 13212–13217. 21788494 10.1073/pnas.1103039108 PMC3156214

