# Supplementary material for: Identification of Gene Clusters Associated with Host Adaptation and Antibiotic Resistance in Chinese Staphylococcus aureus Isolates by Microarray-Based Comparative Genomics
Source: PLoS One. 2013 Jan 7;8(1):e53341. doi: 10.1371/journal.pone.0053341 (PMC3538772; doi:10.1371/journal.pone.0053341)
Supplement: Table S1 — Primers of representative genes used for PCR validation. (DOC) [file pone.0053341.s001.doc]

**Table S**1. Primers of representative genes used for PCR validation.

| **Cluster No.** | **Representative Gene** | **Forward primer** | **Reverse primer** |
| --- | --- | --- | --- |
| C1 | SAV1948 | CGTTGACAACAAGTCCAC | ATACTGTCCTTGAGCACC |
| C2 | SAV1979 | ATGGCGAGAAAAGCAAGG | CAGGTAGCACGCATAACG |
| C3 | SAV2022 | CGTGGTGTTGCTGTGTAC | TACATGCGAATTGTCCCC |
| C4 | SAV0398 | TCATAGACACGCCAGGAC | AGGTTCGCCAGTGGTAAC |
| C5 | SAV0791 | AAGGCGTTCTGCCACTAC | TGGTGTTGTTGTGGTTGG |
| C6 | SAV1813 | CATCTCTTGGTTTCGCTG | TACCTGGTTGTGCATACG |
| C7 | SAV1994 | TCGAACATCGGCGAAATG | TGTGACCGTCCGAATGTG |
| C8 | SAV1313 | AAATTGAAAGAAGATGAACAGG | CTTGCTTCTCGATAAAGTCTC |
| C9 | SAV2498 | TGATGAATGATTTGAAAAGC | ACAGAAACAACAATGATTAC |
| C10 | SAV0031 | TTGACCATGAGCGAACAC | TCCCTGATCTCGACTTCG |
| C11 | SAV0052 | AAAACCCTAAAGACACGC | AAAGCCTGTCGGAATTGG |
| C12 | SAV1656 | TGGTTCAGCAGTAAATGG | CGAGTGCTTTCACCTTTG |
| C13 | SAV0902 | TTGGATTGTGGTCGTTTG | GGGCTATATTCGTTTCGG |
